# Influence of perceived parental views of failure on academic resilience among middle school students: a moderated mediation model

**DOI:** 10.3389/fpsyg.2025.1532332

**Published:** 2025-02-17

**Authors:** Wenying He, Dasheng Shi

**Affiliations:** School of Education, Minzu University of China, Beijing, China

**Keywords:** perceived parental views of failure, academic resilience, growth mindset, parental involvement in education, middle school students

## Abstract

Focusing on a sample of middle school students, this study examined the impact of perceived parental views of failure on academic resilience, as well as the mediating role of growth mindset and the moderating role of parental involvement in education. A total of 2,546 Chinese middle school students were recruited from the Beijing, Fujian, and Guizhou regions to complete a questionnaire. The data were analyzed using structural equation modeling (SEM) in SPSS 26.0 and PROCESS. The results indicate that perceived parental positive views of failure significantly and positively predict academic resilience, and this relationship is mediated by growth mindset. Furthermore, parental involvement in education moderates the direct effect within the mediation model. Interestingly, this effect is stronger at high levels of parental involvement in education but is not evident at low levels. Additionally, the moderating effects of specific dimensions of parental involvement in education (emotional, cognitive, and behavioral involvement) differ. Emotional involvement demonstrates the strongest moderating influence, while behavioral involvement shows the weakest. These findings provide theoretical insights and empirical support for developing intervention strategies aimed at enhancing the academic resilience of middle school students through family education.

## 1 Introduction

When faced with academic setbacks, why do some students recover quickly, like a spring, while others are crushed? To explain this phenomenon, scholars have coined the term “resilience” to describe people’s ability to bounce back in the face of life’s adversities. In the field of education, a similar term, “academic resilience,” has been used to describe students’ ability to recover from academic underperformance (Martin and Marsh, 2008; Waxman et al., 2003). Generally speaking, academic resilience is defined as a student’s ability to successfully overcome setbacks, pressures, and challenges in daily learning activities (e.g., poor grades, exam stress, and difficult coursework) (Martin, 2002). Academic resilience is critical for students’ learning outcomes, as there is substantial evidence showing that students with low academic resilience are more likely to experience poor academic performance (Sadoughi, 2018), procrastination (Lin, 2020), burnout (Oyoo et al., 2018), depression (Zhu, 2022), and even suicidal behaviors (Gallagher and Miller, 2018). Therefore, improving students’ academic resilience is crucial, particularly in East Asian countries like China, where academic pressure is immense.

As the second most populous country in the world, with a total population exceeding 1.4 billion, China’s education expenditure made up only 3.6% of its total Gross Domestic Product in 2020 (UNESCO Institute for Statistics, 2020). This discrepancy has led to limited educational resources, which are unevenly distributed across urban and coastal areas (Zhang, 2023). As a result, academic competition has intensified among students who are striving to secure better educational opportunities (Martin and Hau, 2010; Li and Haibin, 2017). This phenomenon is particularly prevalent among Chinese secondary school students, as they must prepare for two significant standardized tests: The first occurs for 9th graders, who face a standardized test upon completing middle school studies. Only those who score above the 50th percentile are admitted to high school, while the others must attend vocational schools (General Office of the Ministry of Education, 2014). After 3 years of high school, 12th graders must prepare for the National College Entrance Exam (Gaokao), a high-stakes test linked to social mobility (Ross and Wang, 2010).

Influenced by the ancient Chinese imperial examination system and Confucian values, standardized test performance is often regarded as the sole indicator of academic success by parents, schools, and society (Li and Haibin, 2017). To be specific, Confucian values, which emphasize diligence, perseverance, and self-discipline, significantly shape societal and parental expectations, fostering a belief that success is achieved solely through hard work and academic excellence. Similarly, the legacy of the imperial examination system, where success was equated with passing rigorous standardized tests to gain social status, continues to influence societal attitudes. These align with the high-stakes, test-oriented approach of the Gaokao system. As such, studying becomes the most important task for every middle school student, particularly given the test-oriented educational approach adopted by many schools, where teachers focus on preparing students for exams, often at the expense of broader learning (Li and Haibin, 2017). A survey conducted by the China Youth Research Center found that Chinese middle school students spend an average of 11 h at school (China Youth Research Center, 2018), much higher than the OECD average of 7.8 h (PISA, 2018). As a result, students have less time for sleep, leisure, and extracurricular activities. In the face of such immense exam pressure and various learning challenges, academic resilience is not only crucial for higher academic performance but also essential for protecting students from physical and mental health issues during their secondary education years.

To promote students’ academic resilience, parental support is indispensable. In China, many parents express a strong desire to be involved in their children’s education. But what can parents do to help their children improve academic resilience? With this question in mind, we focus on a concept that parents often convey to their children in everyday life: their views on failure (i.e., whether failure is considered beneficial or detrimental). Within the framework of traditional Chinese culture described above, many parents tend to adopt a stricter view of failure, seeing it as a sign of insufficient effort or lack of ability. If this view is internalized by children and failure is stigmatized, will middle school students be less likely to be inclusive of academic failure and, consequently, less resilient? Conversely, might they exhibit higher academic resilience? At the same time, this view of failure seems to share some commonalities with the concept of a “growth mindset” in positive psychology—the belief that intelligence and ability are malleable and can be improved, rather than fixed. Therefore, the four variables of perceived parental views on failure, academic resilience, growth mindset, and parental involvement in education came to our attention, leading to the design and implementation of this study.

Currently, research on the relationship between parents and children’s academic resilience remains limited. Most existing studies have primarily focused on variables such as parenting style (Wolke et al., 2013), parenting skills (Rojas, 2015), parenting consistency (Williams et al., 2022), and parental supervision (Li, 2017) and their effects on children’s academic resilience. However, few studies have specifically examined the relationship between children’s perceived parental views of failure and their academic resilience. To address this gap, this study incorporates two additional variables—growth mindset and parental involvement in education—to further explore the mechanisms underlying the association between perceived parental views of failure and academic resilience. By analyzing survey data, we aimed to develop a moderated mediation model from a family perspective. This model provides empirical support for understanding how perceived parental views of failure influence academic resilience and offers practical suggestions for enhancing academic resilience among middle school students.

### 1.1 Perceived parental views of failure and academic resilience

Children report that academic failures (problems with homework or low grades) are the most common distressing events in their daily experiences (Greene, 1988; Mantzicopoulos, 1990), and parents often share their concern (e.g., Pew Research Center, 2015; Public Agenda, 2011). Children can directly perceive the views of failure conveyed by their parents. According to Haimowitz and Dweck, views of failure can be divided into two types: debilitating versus enhancing. More specifically, those with the former view believe that failure inhibits learning and productivity and must be avoided, whereas those with the latter view believe that failure facilitates learning and growth (Haimovitz and Dweck, 2016). For example, in a typical family scenario in China, a child brings a simple math problem they got wrong to ask for their parent’s help. A parent with a debilitating view might respond with criticism like: “Other kids can do this, but you cannot. I think you just do not have the brain for math, and this will be a problem for your future education.” In contrast, a parent with a enhancing view might say: “It’s okay to make mistakes. Let us think about what part wasn’t clear when you were solving the problem. Was it because you did not fully understand the concept, or did you make a calculation error? Once we figure it out, you will not make the same mistake next time. Finding and correcting mistakes is the biggest gain!”

Studies have shown that parents’ views of their children’s failures largely lead children to construct the same perceptions of failure (Tao et al., 2021). This means that children of parents with an enhancing view of failure also perceive failure as having positive value and believe that they can learn something from it. Based on the modern expectancy-value theory (Eccles and Wigfield, 2002), children who hold an enhancing view of failure are more likely to assign higher value to academic failure experiences and thus have higher levels of academic motivation. The predictive effect of academic motivation on academic resilience has been confirmed by relevant research (Zhao and Yu, 2018; Borman and Overman, 2004). Therefore, it can be hypothesized that perceived parental views of failure are closely related to academic resilience. More specifically, the more positively children perceive their parents’ views of failure, the higher their level of academic resilience is likely to be.

### 1.2 The mediating role of growth mindsets

Growth mindset may be a significant mediating variable in the association between perceived parental views of failure and academic resilience. Growth mindset refers to the belief that intelligence and abilities are malleable and can be improved, rather than fixed and unchangeable (Dweck, 2006). The failure view has been conceptualized as a type of “lay theory”: a guiding, common-sense belief about social phenomena that shapes one’s perceptions of life events, expectations, and behavior (Rippere, 1990). Individuals with an enhancing view of failure hold the belief that failure can lead to growth, including intelligence, ability, courage, and more. This seems to overlap with the definition of a growth mindset. Previous research has also shown that students’ perception of their parents having a debilitating view of failure can lead them to view intelligence as a fixed entity that cannot be changed (Tao et al., 2021). Parents who hold an enhancing view of failure, compared to those with a debilitating view of failure, were more likely to have children who viewed their own intelligence as malleable through personal effort (Haimovitz and Dweck, 2016). Therefore, the more positive the perception of parents’ views of failure, the stronger a child’s growth mindset is likely to be.

Furthermore, growth mindset may exert potential effects on academic resilience. Individuals with a growth mindset do not tend to attribute their intelligence and abilities to innate genetic factors, but rather focus on developing them through challenge-seeking and mastering knowledge (Dweck, 2006). This mindset forms a framework for interpreting and responding to adversity (Molden and Dweck, 2006). When they are in the midst of a setback, they do not view it as indicative of innate inability, but rather as a motivator that drives them to continue learning (Blackwell et al., 2007; Plaks and Stecher, 2007). Thus, they may redouble their efforts, try new strategies, or seek help, and they can maintain their interest and enjoyment (e.g., Blackwell et al., 2007; Hong et al., 1999). Thus, individuals with growth mindsets in various domains (such as intelligence or personality) have shown greater resilience and more adaptive problem-solving following academic and interpersonal setbacks (Burnette et al., 2013). In summary, we could reasonably propose that perceived parental positive views of failure are a facilitator of growth mindset, which would in turn improve students’ academic resilience.

### 1.3 The moderating role of parental involvement in education

Parental involvement in education refers to parental interactions with their children and schools to promote academic success (Hill and Tyson, 2009), such as helping with homework, giving a study reward, and other supportive actions. The resilience dynamic model, proposed by a joint research institution in California, suggests that if external resources can meet certain psychological needs of individuals (such as love, belonging, control, and value), individuals will naturally develop characteristics such as problem-solving, cooperation, and goal orientation. These traits help individuals develop internal resources, protecting them from the influence of adverse factors and enhancing their level of resilience (Du and Zhang, 2020). As one of the key external resources, supportive educational interactions between parents and children (such as assisting with homework and providing rewards) can meet children’s psychological needs and help improve their academic resilience. Previous studies have shown that parental involvement in education significantly improves children’s positive attitudes towards learning, learning perseverance (Sapungan and Sapungan, 2014), self-control (Brody and Flor, 1998), and academic performance (Jeynes, 2012), among other related factors. Although not definitive, these behaviors to a certain extent indicate the positive impact of parental involvement on academic resilience. Other studies have shown that parents’ emotional and academic support, along with a beneficial family environment, can help improve children’s resilience (Miller et al., 2015; Carmona-Halty et al., 2019). Therefore, it can be inferred that parental involvement promotes academic resilience and moderates the relationship between parents’ failure beliefs and academic resilience, as well as between growth mindset and academic resilience.

### 1.4 The present study

**Figure 1:**
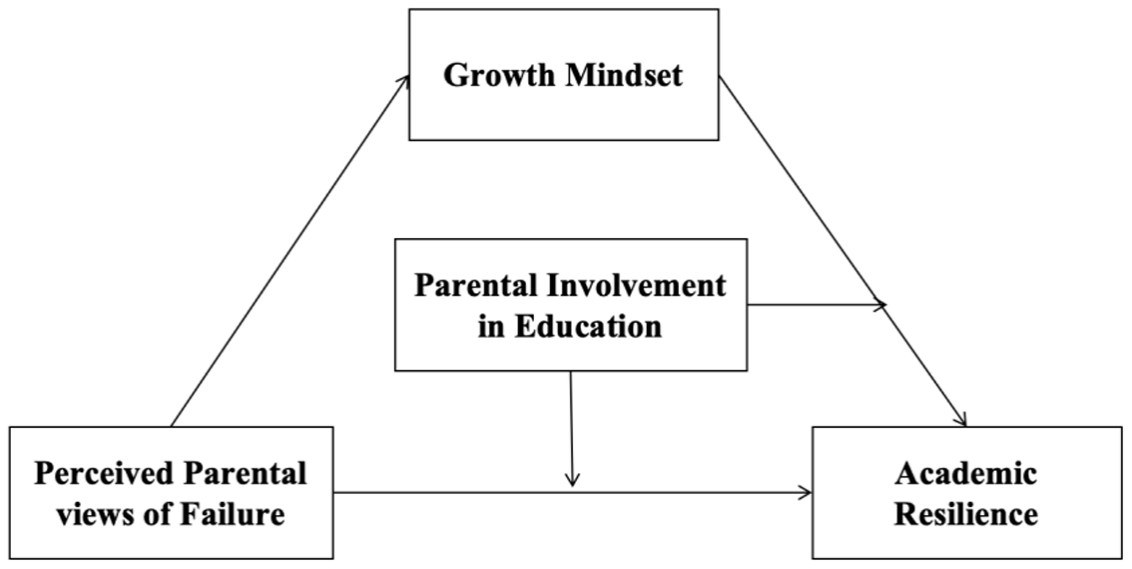
The hypothetical model.

Based on previous literature and theoretical frameworks, the purpose of this study was to explore the relationship between perceived parental views of failure, academic resilience, growth mindset, and parental involvement in education, as well as to establish a moderated mediation model (see Figure 1). This study hypothesized that perceived parental positive views of failure were positively associated with the academic resilience of middle school students in mainland China (H1). Growth mindset would play a mediating role in the association between perceived parental views of failure and academic resilience among middle school students in mainland China (H2). That is, perceived parental positive views of failure may positively predict growth mindset, which in turn may improve academic resilience among these students. Parental involvement in education would play a moderating role in both the direct path and the mediating path of the model (perceived parental views of failure → academic resilience; growth mindset → academic resilience) (H3). The moderating effect will be stronger when a student has a high level of parental involvement in education and weaker when parental involvement is low (H4).

## 2 Method

### 2.1 Participants

In this study, we adopted a cluster sampling method to collect students’ data from 8 middle schools in Beijing, Guizhou, and Fujian provinces between 18 and 30 September 2023 via the Questionnaire Star online platform. To ensure the quality of questionnaire responses and minimize the impact of external pressure on participants, we first explained the purpose and significance of the study, as well as instructions on how to complete the questionnaire. Secondly, participants were informed that there were no right or wrong answers and that they should respond anonymously based on their genuine experiences. Finally, students who volunteered to participate in the study were invited by their teachers to complete the questionnaire in a relaxed and undisturbed setting.

This study was approved by the ethics committee of the author’s institution, and informed consent was obtained from participants, their parents, and the schools for all items. A total of 2,546 questionnaires were distributed, of which 232 were deemed invalid and excluded due to single-option answers, contradictory responses, or extremely short completion times. Thus, 2,314 valid questionnaires were retained, yielding a recovery rate of 90.89%. Of the participants, 1,158 (50.04%) were male, and 1,156 (49.96%) were female. A total of 1,169 participants (50.52%) were in the first year, 904 (39.07%) were in the second year, and 241 (10.41%) were in the third year. The mean age of the students was 13.64 years (SD = 1.34).

### 2.2 Measures

The questionnaire used in this study consisted of 35 items divided into five sections: (a) demographic information, (b) Perceived Parental Views of Failure Scale, (c) Academic Resilience Scale, (d) Growth Mindset Scale, and (e) Parental Involvement in Education Scale. The demographic information section included gender, single-child status, single-parent status, grade, and age. The Perceived Parental Views of Failure Scale, Growth Mindset Scale, and Academic Resilience Scale were originally developed in English. To ensure the equivalence of the scales, three translators used the back-translation method (Brislin, 1970) to translate the scales into Chinese. The translations were then refined and optimized before finalizing the questionnaire.

#### 2.2.1 Perceived parental views of failure scale

The perceived parental views of failure scale, developed by Haimovitz and Dweck (2016), was adopted in this study. It consists of four self-reported items (e.g., “My parents think failure is bad and should be avoided” and “My parents think failure hurts my learning”) that measure the extent to which students perceive their parents as holding a debilitating view of failure. Responses are rated on a 6-point Likert scale (1 = strongly disagree to 6 = strongly agree). In the present study, the scale demonstrated acceptable internal consistency, with a Cronbach’s alpha coefficient of 0.761.

#### 2.2.2 Academic resilience scale

The study used the Academic Resilience Scale, developed by Martin and Marsh (2006, 2008), with four items (e.g., “I think I’m good at dealing with schoolwork pressures (e.g., bad marks, negative feedback on my work)”). It measures the level of students’ academic resilience on a 5-point Likert scale (1 = strongly disagree to 5 = strongly agree). This study found that the scale had good internal consistency, with a Cronbach’s alpha coefficient of 0.806.

#### 2.2.3 Growth mindset scale

The Mindset Scale, developed by Dweck (1999), was used in this study. It consists of six self-report items, with the first three questions measuring fixed mindset (e.g., “Intelligence cannot be changed”) and the last three questions measuring growth mindset (e.g., “It is possible to improve my intelligence”). To measure participants’ level of growth mindset, we used a 5-point Likert scale (1 = strongly disagree to 5 = strongly agree) and reverse-scored the first three items to account for the fixed mindset. Therefore, a higher total score represents a higher level of growth mindset. In the present study, the scale had a Cronbach’s alpha coefficient of 0.728.

#### 2.2.4 Parental involvement in education scale

The study used the Chinese version of the Parental Involvement in Education Scale, originally proposed by Grolnick and Slowiaczek (1994) and later adapted to the Chinese cultural context by Mei et al. (2019). The scale consists of 16 items that cover three dimensions: behavioral involvement (e.g., “My parents ask and supervise my homework”), emotional involvement (e.g., “My parents encourage me when I do badly on the test”), and cognitive involvement (e.g., “My parents buy books and listen to lectures on education”). The scale measures the level of parental involvement in education using a 5-point Likert scale (1 = never to 5 = always). This study revealed satisfactory internal consistency for the total scale (Cronbach’s α = 0.883) and sub-dimensions, with values of 0.764, 0.883 and 0.749, respectively.

## 3 Results

The analyses were conducted using SPSS 26.0 (IBM Corp, 2015) and Hayes’s PROCESS version 4.0. First, descriptive statistics were used to calculate the SD and mean levels of the main variables, and Pearson’s correlations were used to assess the relationships among perceived parental views of failure, growth mindset, parental involvement in education and academic Resilience. Then, all these variables were mean-centered prior to the analyses. Next, model 4 of PROCESS examines the mediating effect of growth mindset and model 15 of PROCESS was used to test whether Parental Involvement in Education could moderate the mediation models. Finally, We propose a modified model which based on the obtained research data.

### 3.1 Common method variance analysis

Since all data for this study were obtained from the self-reports of middle school students, there was a possibility of common method bias, indicating that common method bias should be examined. We used the Harman single-factor test (Harman, 1976) on the present data. The results showed that a total of six factors had eigenvalues greater than one, and the first factor explained 28.720% of the total variation, which is below the 40% threshold criterion (Podsakoff et al., 2003), indicating that no significant common method bias was found.

### 3.2 Descriptive and correlation analyses

Table 1 shows the means, standard deviations (SD), and Pearson’s correlations of the variables. The results suggested significant correlations between parental involvement in education and age, gender, single parent status, and single child status. Single parent status was also significantly associated with perceived parental views of failure, academic resilience, and parental involvement in education, suggesting that subsequent analyses should consider age, gender, single child status, and single parent status as control variables. Additionally, positive correlations existed between perceived parental views of failure, academic resilience, growth mindset, and parental involvement in education, with r values ranging from 0.246 to 0.582.

**Table 1:** The mean (M), standard deviation (SD), and correlations of the variables (*n* = 2,286).

Variable	*M*	SD	1	2	3	4	5	6	7	8
1.Age	13.638	1.337	1							
2.Gender	0.501	0.500	0.028	1						
3.Single child status	0.771	0.420	0.113**	0.043*	1					
4.Single parent status	0.888	0.315	−0.039	−0.032	0.163**	1				
5.Perceived parental views of Failure	4.246	1.056	−0.039	0.011	−0.026	0.053*	1			
6.Academic resilience	4.632	0.958	0.030	−0.026	−0.019	0.066**	0.246**	1		
7.Growth mindset	4.939	0.784	−0.010	−0.006	−0.016	0.034	0.286**	0.582**	1	
8.Parental Involvement in education	4.035	0.947	−0.146**	−0.087**	−0.087**	0.090**	0.258**	0.427**	0.453**	1

**p < 0.01, *p < 0.05. All tests were two-tailed. Gender: male = 0, female = 1; only child = 0, non-only child = 1; single parent = 0, non-single parent = 1; all values are rounded to three decimal places.

### 3.3 Mediation analysis of growth mindset

The bootstrap method was employed to test the confidence intervals (CI) for the proposed model, with 5,000 resampling iterations and Model 4 selected for analysis. The results revealed that the 95% confidence intervals for both the direct and indirect effects did not include 0, indicating statistical significance. Specifically, the total effect was 0.222, with the direct effect accounting for 0.078 and the indirect effect for 0.144. Notably, the indirect effect was responsible for 64.865% of the total effect, which suggests that the growth mindset partially mediates the relationship between parental failure perception and academic resilience (see Table 2). This indicates a mechanism through which perceived parental views of failure may shape students’ academic resilience, primarily by influencing their growth mindset. The incomplete mediation observed suggests that while the growth mindset is an important factor, other unexamined variables may also contribute to the development of academic resilience. These results underscore the critical role of growth mindset in fostering resilience among students, particularly in the context of perceived parental views of failure, and suggest directions for further research into other potential mediators.

**Table 2:** Total effect, direct effect and indirect effect among the variables (standardized coefficient).

	Effect size	Boot SE	Boot CI Lower limit	Boot CI Upper limit	Relative effect size
Total effect	0.222	0.018	0.186	0.258	
Direct effect	0.078	0.016	0.047	0.110	35.135%
Indirect effect	0.144	0.013	0.121	0.170	64.865%

### 3.4 Moderated mediation effects

**Figure 2:**
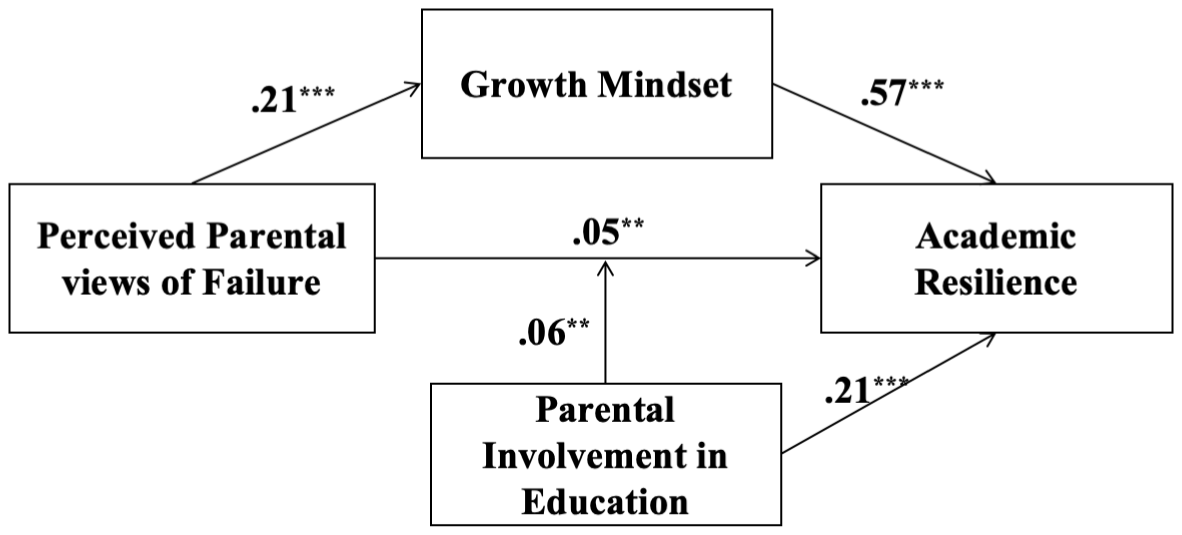
The moderated mediation model.

We used Model 15 of PROCESS to determine whether the mediation effect could be moderated by parental involvement in education. As shown in Table 3, perceived parental views of failure positively predicted growth mindset (*β* = 0.212, *p* < 0.001) and academic resilience (*β* = 0.053, *p* < 0.001). Growth mindset was also a significant positive predictor of academic resilience after controlling for gender, age, and two other factors. In addition, the interaction between perceived parental views of failure and parental involvement in education positively predicted academic resilience (*β* = 0.059, *p* < 0.001), indicating that parental involvement moderated the direct path of the mediated model. However, the interaction between growth mindset and parental involvement in education was not significant (*β* = −0.021, *p* > 0.05), indicating that parental involvement does not moderate the latter half of the path of this mediated model. The verified mediated mediation model is shown in Figure 2.

**Table 3:** Results obtained in the testing of the moderated mediation model (standardized coefficient).

Regression equation		Significance of the regression		Coefficient	Fit index	
Result variable	Predictor variable	*β*	*t*	*95%CI*	*R^2^*	*F*
Growth mindset (M)	Gender	−0.013	−0.402	[−0.074, 0.049]	0.083	41.014^***^
	Age	0.002	0.012	[−0.021, 0.025]		
	Single child status	−0.022	−0.578	[−0.097, 0.053]		
	Single parent status	0.052	1.019	[−0.048, 0.151]		
	Perceived parental views of failure (X)	0.212	14.173	[0.182, 0.244]		
Academic resilience (Y)	Gender	−0.015	−0.460	[−0.077, 0.048]	0.385	158.022^***^
	Age	0.050	4.138	[0.026, 0.073]		
	Single child status	−0.010	−0.265	[−0.086, 0.065]		
	Single parent status	0.100	1.866	[−0.005, 0.195]		
	Perceived parental views of failure (X)	0.053	3.373	[0.022, 0.084]		
	Growth mindset (M)	0.573	24.396	[0.527, 0.619]		
	Parental involvement in education (W)	0.207	10.656	[0.169, 0.245]		
	X × W	0.059	3.647	[0.027, 0.090]		
	M × W	−0.021	−1.000	[−0.061, 0.020]		

To further explore the moderating effects of different parental involvement styles in the mediating model of perceived parental views of failure, growth mindset, and academic resilience, we examined the moderating effects of the three dimensions of parental involvement in education (emotional involvement, cognitive involvement, and behavioral involvement) using the same approach as above. The results showed that all three dimensions moderated the direct effects of the mediation model, with beta coefficients of 0.04 (*p* < 0.001), 0.04 (*p* < 0.001), and 0.03 (*p* < 0.001) for emotional involvement, cognitive involvement, and behavioral involvement, respectively. However, none of the moderating effects on the latter half of the mediation model were significant, consistent with the overall test results above.

**Figure 3:**
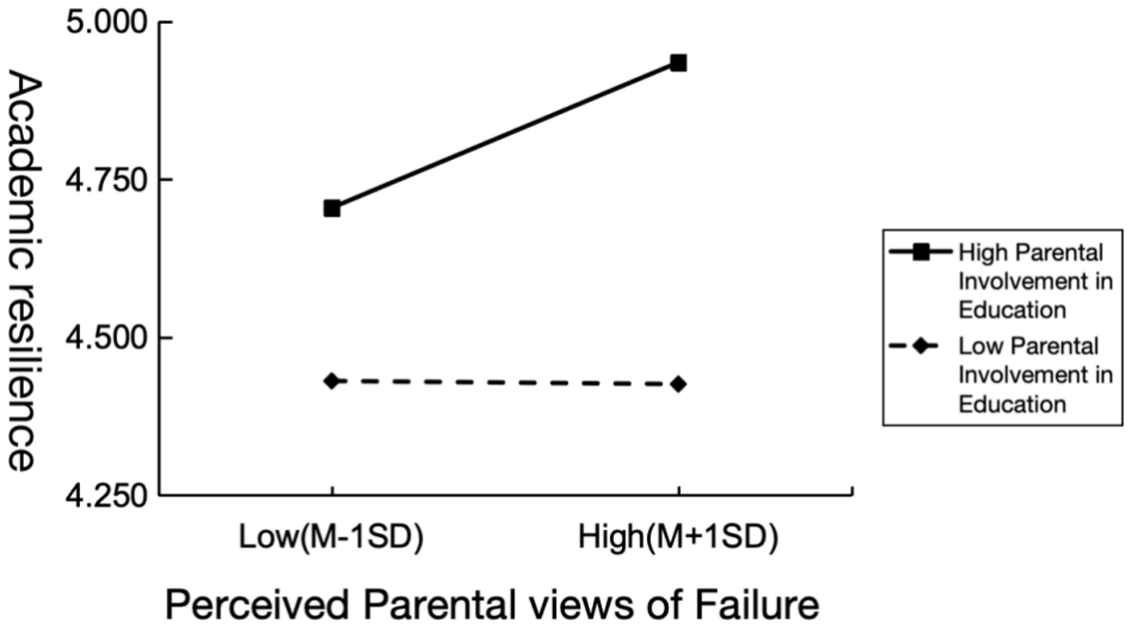
The moderating effect of parental involvement in education on the influence of perceived parental views of failure on academic resilience.

**Figure 4:**
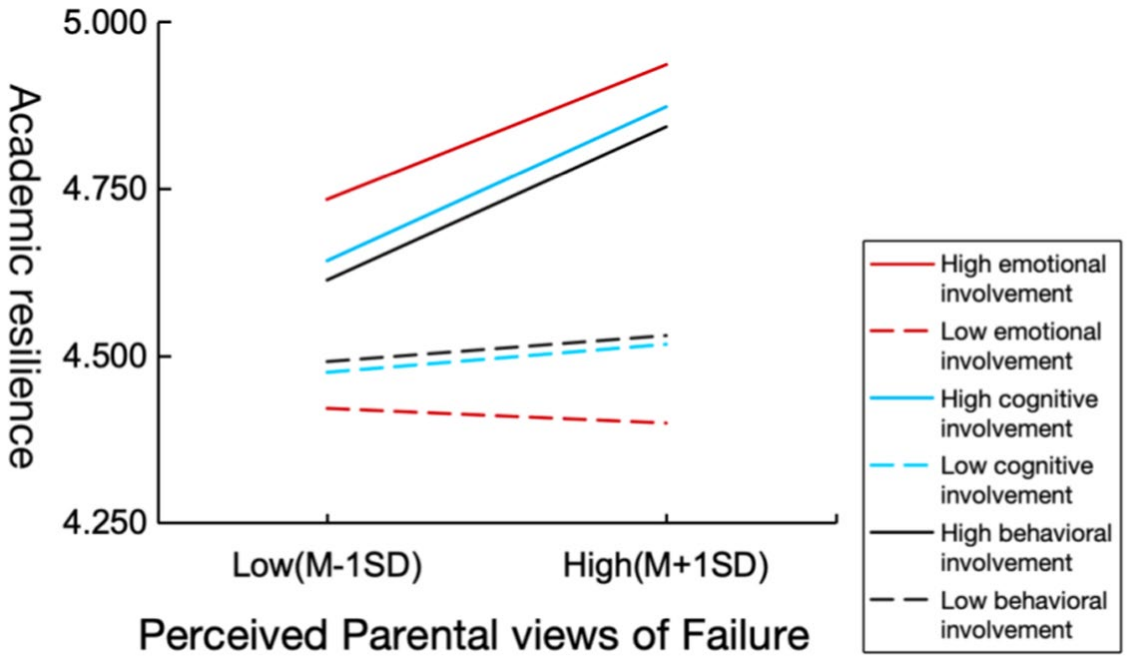
The moderating effect of specific dimensions of parental involvement in education on the influence of perceived parental views of failure on academic resilience.

To visually demonstrate the moderating effect of parental involvement in education on the relationship between perceived parental views of failure and academic resilience, parental involvement in education and its three dimensions were divided into two levels: one standard deviation below the mean (M – 1 SD) and one standard deviation above the mean (M + 1 SD). The moderation effects were illustrated in Figures 3, 4. Simple slope analysis revealed that academic resilience in students with high levels of parental involvement (M + 1 SD) increased as perceived parental views of failure became more positive. Among the three dimensions of parental involvement, emotional involvement was most strongly associated with facilitative effect, followed by cognitive involvement, while behavioral involvement demonstrated the weakest association.

In contrast, the academic resilience of students with low levels of parental involvement (M − 1SD) was barely affected by perceived parental views of failure. Specifically, the effects of low parental cognitive and behavioral involvement were almost identical, with students showing only a slight increase in academic resilience as perceived parental views of failure became more positive. However, students with low parental emotional involvement showed a slight decrease in academic resilience.

## 4 Discussion

Based on previous research and related theories, this study constructs a moderated mediation model. This model not only clarifies the question of ‘How perceived parental views of failure affect academic resilience’ but also addresses the question of under what conditions perceived parental views of failure have a more significant impact on academic resilience. This study has specific theoretical significance for expanding the understanding of the relationship between family factors and academic resilience.

### 4.1 The association between perceived parental views of failure and academic resilience

The present study shows that perceived parental views of failure significantly and positively predicted academic resilience in middle school students, which was consistent with our hypothesis 1. This finding broadens research in the area of academic resilience. According to ecosystem theory (Bronfenbrenner, 1993), individual development is the result of the interaction between an individual and the system in which he or she lives. The family is the innermost micro system in the hierarchy of the environment in which middle school students live and students have direct interaction in it. Therefore, the development of academic resilience of middle school students is closely related to family interactions. Evolutionary Theory of Socialization (Belsky et al., 1991) suggests that parenting behaviors lead children to develop different behavioral patterns to adapt to their environment, and that the more perceived parental positive views of failure through their parents’ behaviors, the more likely they are to develop positive coping behavioral patterns, such as exploring their own resources and external resources to combat setbacks. These behavioral patterns increased academic resilience.

### 4.2 Mediating effects of growth mindset

According to the results of the mediation analysis, growth mindset mediated the association between perceived parental views of failure and academic resilience. More specifically, perceived parental positive views of failure were a positive predictor of growth mindset, which, in turn, improved academic resilience. This finding was consistent with our hypothesis 2. To the best of our knowledge, this full model of the relationships between perceived parental views of failure, growth mindset, and academic resilience has never been tested. However, two of the paths have been examined separately, and the causal relationships have been established. In the literature, research has found that (a) more positive perceived parental views of failure lead to higher growth mindset (Tao et al., 2021; Haimovitz and Dweck, 2016); and (b) higher growth mindset contributes to resilience (Yeager and Dweck, 2012). The current findings support previous studies as well as the organizational framework for conceptualizing resilience in children proposed by Mandleco (2000).

After introducing the mediating variable of growth mindset, we found that the effect value of perceived parental views of failure on academic resilience changed significantly (from 0.222 to 0.078), indicating that family-internal factors (perceived parental views of failure) and psychological factors (growth mindset) indeed interact or have a transactional relationship that affects resilience. From a social-constructivist perspective (Grolnick and Slowiaczek, 1994), when students perceive that their parents hold an enhancing view of failure, they may also acquire similar positive attitudes towards failure through interactive socialization processes. These children believe that failure is not a bad thing but rather an opportunity for growth, thereby naturally developing a growth mindset. They are more likely to attribute failure to a lack of effort rather than to a lack of ability (Blackwell et al., 2007; Henderson and Dweck, 1990), seeing it as a chance for self-improvement. This perspective generates greater motivation to pursue success, a stronger interest in challenges, and greater determination to overcome failure (Rhew et al., 2018). Thus, these children demonstrate higher levels of academic resilience.

### 4.3 Moderating effect of parental involvement in education

The present study found that parental involvement in education promotes the effect of perceived parental views of failure on academic resilience, but the effect of growth mindset on academic resilience was not influenced. This partially confirms H3. This finding indicates that the influence of growth mindset on academic resilience may involve a complex psychological mechanism of individual self-internalization, which is neither facilitated nor inhibited by parental involvement in education. However, parental involvement had a positive effect on individual academic resilience (*β* = 0.21).

In addition, the findings showed that the effect of perceived parental views of failure on academic resilience was more significant for middle school students with higher levels of parental involvement in education compared to those with lower levels. This result was consistent with our hypothesis 4. According to ecological systems theory, individual development results from the interaction between the individual and the systems they are embedded in, and this interactive process requires maintaining a certain frequency and duration over time (Bronfenbrenner, 1993). High levels of parental involvement imply that parents and children engage in more frequent and longer interactions. Therefore, these parents are more likely to be involved in their children’s academic failures, such as comforting them when they fail a test or assisting them with difficult homework. Through these interactions, parents convey their attitudes toward failure, including specific academic failures. Middle school students with higher levels of parental involvement in education are thus more likely to have a clearer perception of their parents’ views on failure. As a result, the clearer the perception of parents’ enhancing view of failure, the higher the student’s academic resilience is likely to be.

Surprisingly, when parental involvement in education was low, there was little change in students’ academic resilience despite the increasing degree of perceived parental enhancing views of failure. This suggests that low levels of parental involvement in education had negligible moderating effects. It can be speculated that there may be a threshold requirement for the occurrence of moderating effects, such as a minimum frequency or duration of interactions. In other words, the positive predictive effect between the perception of parents’ positive view of failure and students’ academic resilience may only manifest above a certain level of parental involvement in education.

Moreover, examining the specific dimensions of parental involvement in education revealed only slight differences in the moderating effects of cognitive involvement and emotional involvement. In the high-scoring group, emotional involvement had the greatest promoting effect, followed by cognitive involvement, while behavioral involvement had the smallest effect. In the low-scoring group, the opposite trend was observed, with emotional involvement showing the smallest impact and even a slight inhibitory effect. This suggests that emotional involvement is a more sensitive moderating factor, as it not only maximally enhances academic resilience but also influences the relationship between the two variables either positively or negatively. Compared to cognitive and behavioral involvement, parental emotional involvement better meets children’s needs for belonging and love. According to the resilience dynamic model proposed by a joint research institution in California, meeting more psychological needs is more likely to promote the development of individual academic resilience (Du and Zhang, 2020). Therefore, parental support in the emotional aspects of their children’s academics is particularly crucial.

### 4.4 Implications

Theoretically, our study links perceived parental views of failure with academic resilience, providing novel insights that deepen the understanding of the positive impact of family systems on the mechanisms of academic resilience. Additionally, the researchers analyzed mediating and moderating effects. Specifically, growth mindset was found to mediate the relationship between perceived parental positive views of failure and academic resilience. Perceived parental positive views of failure increased students’ growth mindset, which in turn positively influenced academic resilience. Meanwhile, parental involvement in education moderated the relationship between perceived parental views of failure and academic resilience.

Practically, this study describes the relationships among the four variables examined, offering insights that may help researchers better understand the mechanisms through which academic resilience develops in response to perceived parental views of failure. Furthermore, it provides important implications for parents aiming to promote academic resilience in middle school students. Parents play a critical role in shaping how middle school students perceive failure, making it essential to raise and enhance parents’ awareness of passing on positive views of failure to their children.

On the one hand, parents should engage in more educational interactions with their children, particularly in emotional aspects, such as providing timely support, comfort, and encouragement when children experience academic failures. These interactions need to be maintained at a certain frequency and duration. In addition to face-to-face communication, parents should leverage various means of communication, such as mobile phones and computers, to stay connected with their children when living apart or otherwise unable to be physically present.

On the other hand, parents should enhance their communication skills to offer advice in a more constructive and positive manner, ensuring that children effectively perceive and internalize these messages. In doing so, parents should avoid overreacting in either a positive (e.g., excessive comfort or sympathy) or negative (e.g., punishment or disappointment) direction when responding to their children’s academic failures. Excessive comfort may trigger the boomerang effect (Brummelman et al., 2014), as sympathy can convey the unintended message that failure reflects low ability, leading children to attribute underperformance to a lack of capability. Similarly, expressing disappointment may also communicate a detrimental view of failure. Therefore, parents should guide children to understand academic setbacks objectively, adopt a calm attitude to reduce the perceived harm of failure, and emphasize the value of effort and learning from experience.

Additionally, schools and educational departments should actively provide specific training programs for parents on communication skills, such as online courses, community lectures, or parent-teacher meetings. These programs can help parents better understand the messages they convey to their children during moments of academic failure and ensure these interactions foster resilience.

### 4.5 Limitations and future study

There are several limitations to this study. Firstly, the use of a cross-sectional design, while effective in identifying correlations between variables, does not allow for causal inferences. Future researchers could conduct longitudinal studies to assess whether the measured scores effectively represent actual increases in academic resilience over time. Secondly, this study treated academic resilience as a single, undifferentiated variable, without considering the potential influence of its specific dimensions. Future research should explore whether different dimensions of academic resilience are influenced by perceived parental views of failure through distinct mechanisms. Thirdly, this study demonstrated the moderating effect of parental involvement in education on the relationship between perceived parental views of failure and academic resilience. However, significant differences in the moderating effects were observed based on varying levels of parental involvement, particularly in emotional involvement, which was shown to impact the relationship between the two variables either positively or negatively. Future research could conduct more detailed analyses of this variable to determine whether specific threshold criteria exist for the occurrence and modulation of moderating effects. Finally, the use of self-reported data may introduce sample bias, potentially diminishing the validity of the findings. To reduce biases and enhance the reliability of future research, it is recommended to employ multiple data collection methods, such as third-party observations or triangulation.

## 5 Conclusion

This study found the following: (a) Perceived parental views of failure have a significant impact on the academic resilience of middle school students. When parents adopt a more positive attitude toward failure, middle school students exhibit higher levels of academic resilience. (b) Growth mindset partially mediates the relationship between perceived parental views of failure and academic resilience. This indicates that perceived parental views of failure not only directly influence academic resilience but also exert an indirect influence through growth mindset. (c) Parental involvement in education moderates the relationship between perceived parental views of failure and academic resilience, specifically moderating the direct pathway in the mediation model. Under high levels of parental involvement in education (M + 1 SD), students’ academic resilience increases significantly with their parents’ positive attitudes toward failure. However, under low levels of parental involvement in education (M – 1 SD), students’ academic resilience is almost unaffected by their parents’ attitudes toward failure. (d) The moderating effects of specific dimensions of parental involvement in education vary. In the high-involvement group (M + 1 SD), emotional involvement has the strongest promoting effect, followed by cognitive involvement, with behavioral involvement having the smallest effect. Conversely, in the low-involvement group (M – 1 SD), the effects are reversed, with emotional involvement showing the least impact and even exhibiting a slight inhibitory effect.

## Data Availability

The raw data supporting the conclusions of this article will be made available by the authors, without undue reservation.
